# *ErbB2*-intronic MicroRNA-4728: a novel tumor suppressor and antagonist of oncogenic MAPK signaling

**DOI:** 10.1038/cddis.2015.116

**Published:** 2015-05-07

**Authors:** D C Schmitt, L Madeira da Silva, W Zhang, Z Liu, R Arora, S Lim, A M Schuler, S McClellan, J F Andrews, A G Kahn, M Zhou, E-YE Ahn, M Tan

**Affiliations:** 1 Department of Oncological Sciences, Mitchell Cancer Institute, University of South Alabama, 1600 Springhill Ave, Mobile, AL 36604, USA; 2 Department of Medical Laboratory Science, Xiangya School of Medicine, Central South University, Changsha 410013, China; 3 Cancer Research Institute, Central South University, Key Laboratory of Carcinogenesis, Ministry of Health, Key Laboratory of Carcinogenesis and Cancer Invasion, Ministry of Education, Changsha 410078, China; 4 Department of Comparative Medicine, University of South Alabama, 307 N. University Blvd, Mobile, AL 36688, USA; 5 Department of Pathology, University of South Alabama, 307 N. University Blvd, Mobile, AL 36688, USA; 6 Hunan Cancer Hospital and the Affiliated Tumor Hospital of Xiang-Ya School of Medicine, Central South University, Changsha 410013, China; 7 Department of Biochemistry and Molecular Biology, University of South Alabama, 307 N. University Blvd, Mobile, AL 36688, USA

## Abstract

Although the role of the *ErbB2/HER2* oncogene in cancers has been extensively studied, how ErbB2 is regulated remains poorly understood. A novel microRNA, mir-4728, was recently found within an intron of the *ErbB2* gene. However, the function and clinical relevance of this intronic miRNA are completely unknown. Here, we demonstrate that mir-4728 is a negative regulator of MAPK signaling through directly targeting the ERK upstream kinase MST4 and exerts numerous tumor-suppressive properties *in vitro* and in animal models. Importantly, our patient sample study shows that mir-4728 was under-expressed in breast tumors compared with normal tissue, and loss of mir-4728 correlated with worse overall patient survival. These results strongly suggest that mir-4728 is a tumor-suppressive miRNA that controls MAPK signaling through targeting MST4, revealing mir-4728's significance as a potential prognostic factor and target for therapeutic intervention in cancer. Moreover, this study represents a conceptual advance by providing strong evidence that a tumor-suppressive miRNA can antagonize the canonical signaling of its host oncogene.

Breast cancer is a major health problem in the United States, accounting for over 232 000 new diagnoses and nearly 40 000 fatalities in 2013.^1^ Deregulation of microRNAs (miRNAs) has been implicated in the progression of breast cancer.^2^ MiRNAs are small, non-coding RNA molecules capable of silencing gene expression by binding with complementary targets to cause translational repression or direct mRNA degradation. Therefore, depending on their target genes, miRNAs can play tumor-suppressive or oncogenic roles.

The human epidermal growth factor receptor 2 gene (*ErbB2/HER2,* hereafter called *ErbB2*), encodes a 185-kDa transmembrane protein that belongs to the epidermal growth factor receptor family.^3, 4^ Through its downstream signaling pathways, such as the mitogen-activated protein kinase (MAPK) pathway, ErbB2 regulates several important cell functions in cancer development and progression, such as growth, differentiation, and apoptosis.^5^ The *ErbB2* gene is amplified or overexpressed in approximately 25% of human breast carcinomas and plays a role in many other human malignancies.^6, 7^

Introns, originally thought to be nonsense spacing elements in gene structure, have received attention in recent years owing to the discovery of important functions for these sequences. However, the mechanisms by which intronic miRNAs regulate oncogenes or tumor-suppressor genes and the roles of intronic miRNAs in cancer development and progression are poorly understood. In 2011, by next-generation sequencing techniques, mir-4728 was found to be encoded within an intron of the *ErbB2* gene.^8^ The discovery of mir-4728 within an intron of *ErbB2* has led to new questions regarding the regulation of ErbB2 signaling. Therefore, it is important to determine what role this miRNA plays in human cancers.

In this study, we investigated the role of mir-4728 in breast cancer and its underlying mechanism. We demonstrated a critical role of mir-4728 in the regulation of MAPK signaling and breast cancer tumorigenesis. Our results indicate that mir-4728 is a novel tumor-suppressive miRNA in breast cancer that can not only potentially serve as a biomarker for breast cancer progression and as a future target for therapeutic intervention, but also represents a novel class of antagonistic intronic miRNAs that has remained elusive to researchers.

## Results

### mir-4728 decreases the growth and metastatic potential of breast cancer cells

**Figure 1:**
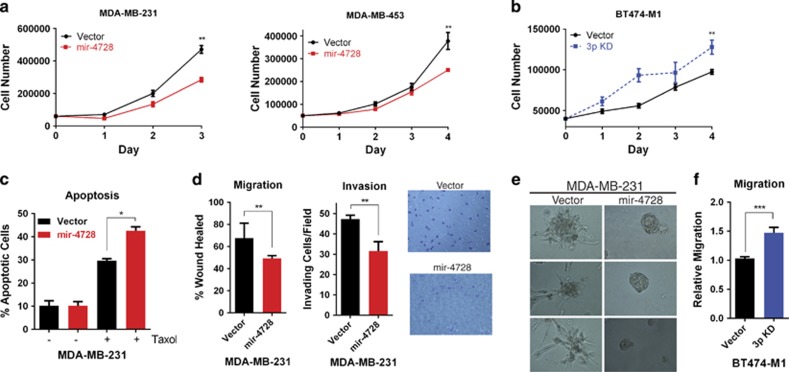
Ectopic expression of mir-4728 affects cell growth, apoptosis, migration, invasion, and metabolism. (**a**) Stable cell lines overexpressing mir-4728 precursor or control vector were created from MDA-MB-231 and MDA-MB-453 cells, and cell proliferation was determined by direct cell counting. (**b**) Stable cell lines with specific miR-4728-3p knockdown or control vector were created from BT474-M1 cells, and cell proliferation was determined by direct cell counting, (**c**) mir-4728 sensitizes breast cancer cells to Taxol-induced apoptosis, (**d**) Wound-healing scratch assays and Matrigel-modified Boyden chamber assays, respectively. Representative images are shown for the invasion assay. (**e**) 3-D colonies grown for 8 days in Matrigel comparing MDA-MB-231 cells stably expressing vector control or mir-4728 overexpression plasmid. (**f**) Specific knockdown of miR-4728-3p increases cell migration in Boyden chamber assays. All error bars indicate±S.E.M. A *P* value of <0.05 is indicated by *, *P*<0.01 by ** and *P*<0.001 by ***

From the mir-4728 precursor, two mature miRNAs are formed, miR-4728-3p and -5p. Of these, miR-4728-3p is the predominantly expressed mature miRNA.^8^ To examine the role of mir-4728 in breast cancer growth and metastasis, we first screened eight breast cancer cell lines to determine endogenous miR-4728 expression (Supplementary Figure 1). We then overexpressed mir-4728 in the breast cancer cell lines MDA-MB-231 and MDA-MB-453, which express relatively low levels of endogenous mir-4728. The expression level of the predominant form of mir-4728, miR-4728-3p, in our overexpression models was comparable with the level present in natural mir-4728 high-expressing breast cancer cell lines (Supplementary Figure 2). In both MDA-MB-231 and MDA-MB-453 cells, overexpression of mir-4728 significantly decreased the rate of cell proliferation (Figure 1a). In contrast, inhibition of miR-4728-3p in the breast cancer cell line BT474-M1, which expresses high levels of endogenous miR-4728-3p, was sufficient to increase cell proliferation (Figure 1b). Additionally, we found that overexpression of mir-4728 increased Taxol-induced apoptosis, as detected by flow cytometry analysis using Annexin V/Propidium Iodide staining (Figure 1c). These findings indicate that mir-4728 decreases the growth and metastatic potential of breast cancer cells, and mir-4728 may sensitize breast cancer cells to Taxol treatment.

We next examined the role of miR-4728 in cancer cell migration and invasion. Expression of mir-4728 decreased the migration potential of MDA-MB-231 cells in wound-healing assays as well as decreased their ability to invade through a Matrigel matrix in Boyden chamber invasion assays (Figure 1d). As all migration and invasion assays were performed using a timeframe of less than 24 h, and mir-4728 exerts little to no effect on cell proliferation at this time point (Figure 1a), this suggests that mir-4728 is capable of independently suppressing the intrinsic invasive potential of cancer cells.

To further examine the suppression of the invasive phenotype, we performed 3-dimensional (3-D) colony growth assays. Comparing MDA-MB-231 cells stably expressing either vector control plasmid or mir-4728 overexpression plasmid revealed that mir-4728 expression suppressed invasiveness in a 3-D environment. Cells expressing mir-4728 formed typical spheroids, whereas the cells expressing the control vector alone showed a distinct invasive phenotype with multiple cells breaking free from the 3-D structures (Figure 1e).

In support, specific knockdown of endogenous miR-4728-3p in mir-4728 high-expressing BT474-M1 cells was sufficient to increase the migration potential of cancer cells (Figure 1f). Taken together, these data indicate that miR-4728-3p inhibits cancer cell proliferation, migration, and invasion, and also induces apoptosis, thereby exerting multiple tumor-suppressor properties.

### mir-4728 negatively regulates MAPK signaling

As previous reports have shown that some intronic miRNAs can directly target their host,^9, 10^ we studied whether mir-4728 could function as a negative feedback mechanism by directly targeting its host gene, *ErbB2*. First, we used the miRNA target prediction algorithms targetscan.org^11^ and mirdb.org^12^ to determine whether *ErbB2* was a predicted target of miR-4728-3p, but found that it was not. To confirm that miR-4728-3p does not directly alter ErbB2 protein levels or activity, we both overexpressed and knocked down miR-4728-3p in ErbB2-expressing cells and examined total ErbB2 expression and phosphorylation status. We found that neither miR-4728-3p overexpression nor knockdown altered ErbB2 protein level or activity (Supplementary Figure 3).

**Figure 2:**
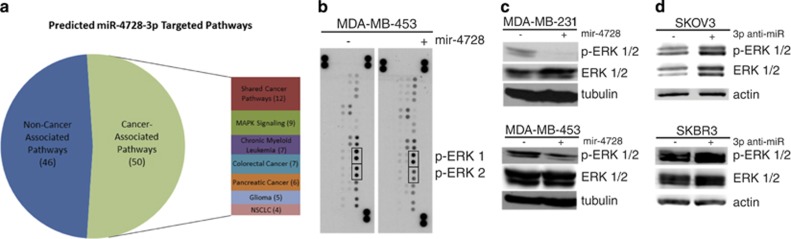
mir-4728 decreases MAPK signaling. (**a**) Genes associated with cancer signaling pathways represent a large portion of predicted miR-4728-3p targets by KEGG pathways analysis. (**b**) mir-4728 overexpression decreases the phosphorylation of several human kinases, most significantly p-ERK 2. (**c**, **d**) Western blots following mir-4728 overexpression or specific miR-4728-3p knockdown, respectively

Using the miR-4728-3p predicted target list generated by targetscan.org, we used the DAVID^13, 14^ bioinformatics tool for functional annotation to input the top-ranked predicted targets for miR-4728-3p. We then used KEGG Pathway^15^ mapping to identify pathways containing genes targeted by miR-4728-3p. Strikingly, of the 96 genes in the output, 50 were involved in cancer-associated pathways (Figure 2a), and 9 were associated with the MAPK signaling pathway. Therefore, we examined the possibility that mir-4728 could affect ErbB2 downstream signaling, specifically the MAPK pathway. After isolating protein lysates from control and mir-4728-overexpressing cancer cells, we used a human phospho-kinase array to analyze the phosphorylation status of 24 different kinases, including MAPKs, ERK 1/2, JNK1-3, p38 isoforms, AKT, and others. Many of these are important molecules of ErbB2 downstream signaling pathways that mediate cell proliferation and survival. Interestingly, overexpression of mir-4728 decreased the phosphorylation of several components of the MAPK family, but most significantly reduced ERK 2 phosphorylation by more than 40% (Figure 2b). ERK 1 phosphorylation was also reduced. Subsequent western blot analyses confirmed that mir-4728 overexpression decreased the levels of activated ERK 1/2 in cancer cells (Figure 2c). Inhibiting endogenous miR-4728-3p in miR-4728-3p high-expressing cells with a specific anti-miR was sufficient to increase the levels of p-ERK 1/2 (Figure 2d). These results demonstrate that mir-4728 functions as an endogenous suppressor of ERK.

### miR-4728-3p directly targets the kinase MST4

**Figure 3:**
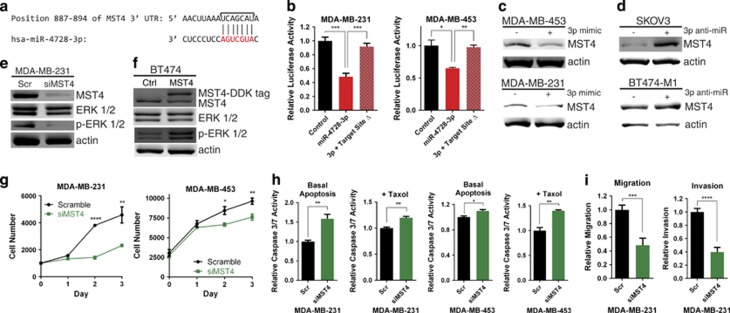
MST4 is a direct target of miR-4728-3p. (**a**) The MST4 3′ UTR contains a predicted miR-4728-3p-binding site. Alignment between the miR-4728-3p seed sequence and MST4 3′ UTR is shown. The bracket indicates the nucleotides deleted from the MST4 3′ UTR for the target site deletion in **b.** (**b**) Dual luciferase reporter assay. Luciferase reporter constructs containing wild-type or target site-deleted MST4 3′ UTRs were cotransfected with control miR or miR-4728-3p mimic into breast cancer cells. Relative firefly luciferase expression was normalized to Renilla luciferase. (**c**, **d**) Western blot showing MST4 protein levels in cells transfected with miR-4728-3p mimic or anti-miR, respectively, for 48 h. (**e**) siRNA against MST4 was transfected into MDA-MB-231 cells and western blot was performed 48 h after transfection. (**f**) MST4 overexpression vector was transfected into BT474 cells and western blot was performed 48 h after transfection. (**g**, **h**, **i**) Breast cancer cells were transfected with MST4 siRNA and proliferation, basal apoptosis, Taxol-induced apoptosis, migration, and invasion were assayed. All error bars indicate±S.E.M. A *P* value of <0.05 is indicated by *, *P*<0.01 by **, *P*<0.001 by ***, and *P*<0.0001 by ****

As mir-4728 suppresses ERK activation, we reasoned that this miRNA may regulate a kinase that is responsible for activation of ERK. To find potential mRNA targets of miR-4728-3p, we screened targetscan.org and mirdb.org for direct targets, starting with the most highly predicted targets and kinases capable of activating ERK. We found that the kinase MST4 contains a predicted miR-4728-3p target site within its 3′ UTR (Figure 3a) that is highly conserved in multiple species (Supplementary Figure 4). MST4, also known as MASK, is a member of the germinal center kinase III subfamily, which belongs to the larger Sterile 20-related family of kinases, which are known to be involved in signaling through MAPK pathways.^16^ MST4 has been shown to regulate multiple cellular aspects, such as cell proliferation and polarity,^17^ and has also been shown to activate ERK as well as contribute to the transformation of Phoenix cells.^16, 18, 19^ Therefore, it is evident that MST4 has certain functions that may aid tumor growth. To determine whether miR-4728-3p was able to directly target the 3′ UTR of MST4, we performed a luciferase assay in which the 3′ UTR of MST4 was fused to a luciferase reporter gene. The presence of miR-4728-3p significantly decreased luciferase activity in breast cancer cells (Figure 3b) as well as in HeLa cells (Supplementary Figure 5). Deletion of the miR-4728-3p target site from the 3′ UTR of MST4 abrogated this effect, demonstrating that this specific site was required for miR-4728-3p binding (Figure 3b and Supplementary Figure 5). To confirm this binding was functionally capable of decreasing MST4 protein expression, western blot analyses were performed. Overexpression of miR-4728-3p decreased MST4 protein levels in breast cancer cells (Figure 3c) as well as in HeLa cells (Supplementary Figure 6) and did so in a dose-dependent manner (Supplementary Figure 7). Furthermore, inhibiting miR-4728-3p with a specific anti-miR in breast and ovarian cancer cells that endogenously express moderate to high levels of miR-4728-3p was sufficient to increase MST4 protein levels (Figure 3d). These results demonstrate that miR-4728-3p directly targets MST4 to inhibit its expression.

We next sought to determine whether the impact of mir-4728 on MAPK signaling was achieved through MST4. We inhibited MST4 with siRNA and examined the effect on ERK activity. After MST4 siRNA transfection, MST4 protein level was decreased and p-ERK 1/2 was also decreased (Figure 3e). We then expressed MST4 in endogenous mir-4728 high-expressing BT474 cells and observed an increase in ERK activity (Figure 3f). These data show that MST4 mediates the impact of miR-4728-3p on MAPK signaling in cancer cells.

To examine whether decreased protein levels of MST4 could explain the tumor-suppressor properties of mir-4728, the assays on cell proliferation, apoptosis, migration, and invasion were repeated. Specific knockdown of endogenous MST4 using siRNA decreased cancer cell proliferation in a manner similar to mir-4728 overexpression, indicating that mir-4728-mediated inhibition of proliferation is, at least in part, through targeting of MST4 (Figure 3g). Similarly, knockdown of MST4 using siRNA also increased the apoptosis of cancer cells under both normal (basal) and stressed (Taxol-treated) conditions (Figure 3h), indicating that induction of apoptosis by mir-4728 may be *via* targeting MST4. Furthermore, knockdown of MST4 also decreased cancer cell migration and invasion (Figure 3i), similar to mir-4728 overexpression. To further support this evidence, rescue of MST4 in mir-4728-overexpressing cells was sufficient to restore the migration potential that was diminished by mir-4728 overexpression (Supplementary Figure 8), demonstrating the importance of the miR-4728-3p/MST4 axis in cancer cell metastasis. Overall, these data demonstrate that the targeting of MST4 by miR-4728-3p is a key step for this miRNA to exert its tumor-suppressive effects.

### mir-4728 inhibits tumor growth in xenograft mouse models

**Figure 4:**
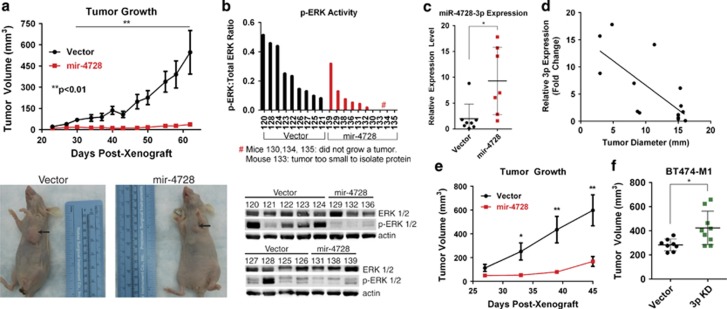
mir-4728 inhibits tumor growth in a xenograft model. (**a**) Tumor growth in nude mice s.c. injected in the mammary fat pad with MDA-MB-231 cells transfected with control plasmid or mir-4728 expression plasmid (*n*=9 for control vector group and *n*=10 for mir-4728 group). (**b**) Phosphorylated ERK protein levels from total protein lysates from the xenograft tumors and ImageJ quantitation of band intensity. (**c**) miR-4728-3p expression from xenograft tumors determined by qPCR. (**d**) miR-4728-3p expression inversely correlated with tumor size as determined by qPCR. Linear regression was performed to fit a line and the equation of the line is Y=−0.8979x+15.62. (**e**) Tumor growth in nude mice s.c. injected in the mammary fat pad with MDA-MB-231 cells identical to the experiment shown in part **a** was again repeated and similar results were observed. (**f**) Specific miR-4728-3p knockdown increases tumor growth. All error bars indicate±S.E.M. A *P* value of <0.05 is indicated by * and *P*<0.01 by **

With our *in vitro* data demonstrating that mir-4728 has important tumor-suppressor properties, we examined whether mir-4728 could suppress tumor growth *in vivo* by injecting mir-4728-overexpressing or vector control MDA-MB-231 cells into the mammary fat pads of 6-week-old female athymic nude mice. Sixty days after tumor cell injection, the average tumor volume in the vector control group reached approximately 500 mm^3^, whereas mir-4728-overexpressing tumors remained very small (less than 50 mm^3^), indicating that mir-4728 drastically decreased tumor growth (Figure 4a). Moreover, at this time point, although all the mice in the vector control group formed tumors, 4/10 mice in the mir-4728-overexpressing group remained tumor-free, indicating that mir-4728 may be able to inhibit tumor formation. After 60 days of tumor growth, we isolated total protein lysate from the tumors and found that overall ERK activity was decreased in mir-4728-overexpressing cells (Figure 4b), which was in agreement with our *in vitro* findings. To determine whether mir-4728 could continue to suppress tumor formation, the mice in the mir-4728-overexpressing group were monitored for 30 additional days. Strikingly, by day 90, 3/10 mice in the mir-4728-overexpressing group remained tumor-free. To ensure that mir-4728 expression had not been lost, we isolated RNA from the tumors and performed real-time qPCR. We found that miR-4728-3p expression had indeed been retained and remained on average approximately ninefold higher than the control vector cells (Figure 4c). Interestingly, we also discovered that expression of miR-4728-3p was strongly inversely correlated with tumor size (Figure 4d), giving additional support to our finding that mir-4728 has tumor-suppressive effects in breast cancer. To validate the striking reduction in tumor growth observed *in vivo*, we repeated the mouse-model experiment to generate an additional tumor growth curve. Again, we found that, after mir-4728 overexpression, breast cancer tumor growth was significantly inhibited in mice (Figure 4e).

To study the effects of loss of mir-4728 expression on tumor growth, we knocked down miR-4728-3p in mir-4728 high-expressing BT474-M1 cells. As expected, knocking down miR-4728-3p caused tumors to grow more rapidly compared with the control vector group. By 3 weeks post xenograft, the difference in tumor formation became statistically significant (Figure 4f). This suggests that miR-4728-3p plays a critical role in inhibiting early tumor growth and further supports our evidence that modulating miR-4728-3p levels has a direct impact on tumor growth *in vivo*.

### Loss of miR-4728-3p expression in breast cancer correlates with worse patient survival

To examine the clinical relevance of our findings, we used *in situ* hybridization to analyze the expression of miR-4728-3p in microarrays containing breast cancer tissue and normal adjacent tissue from human patients. Positive miR-4728-3p expression was detected in 70.7% (29/41) of matched normal adjacent breast tissues, whereas only 35.5% (33/93) breast cancer tissues expressed miR-4728-3p (*P*<0.001). This finding indicates that loss of miR-4728-3p may play a role in human breast cancer development or progression and was consistent with mir-4728's *in vitro* and *in vivo* tumor-suppressive functions. Representative images of normal tissue with positive miR-4728-3p staining and cancer tissue with negative staining are shown in Supplementary Figure 9.

**Figure 5:**
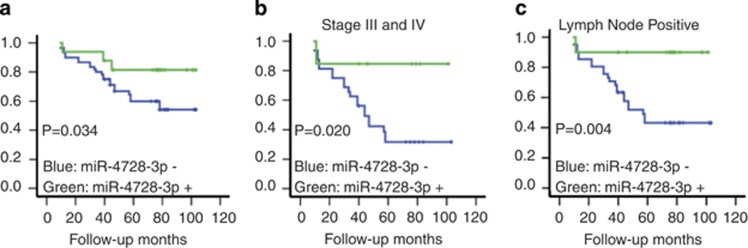
Loss of miR-4728-3p expression occurs in breast cancer and correlates with worse patient overall survival. Human breast cancer TMAs containing matched normal adjacent mammary tissues were used to detect miR-4728-3p expression by *in situ* hybridization. (**a**) Kaplan–Meier survival curves for all cases of breast cancer studied, (**b**) only among stage III and IV cases, and (**c**) only among patients positive for lymph node metastasis

We also studied the correlation of miR-4728-3p expression with patient survival by Kaplan–Meier survival analysis. Breast cancer patients with positive expression of miR-4728-3p had significantly improved overall survival compared with patients who had lost miR-4728-3p expression (Figure 5a). Among stage 3 and 4 breast cancer patients, as well as among patients experiencing lymph node metastases, loss of miR-4728-3p significantly correlated with a worse prognosis (Figures 5b and c), indicating that miR-4728-3p may also play a role in breast cancer metastasis. Additionally, univariate Cox Regression analysis revealed a decreased hazard ratio (HR) for those patients who retained the expression of miR-4728-3p (Table 1), indicating that those patients retaining miR-4728-3p expression are less likely to experience an early death. As internal controls for our human breast cancer tissue microarrays (TMAs), we used the same analysis to show that those patients experiencing lymph node metastasis or advanced stage disease had increased HRs (>1) and were therefore more likely to experience an early death.

We next determined the effect of mir-4728-3p expression on the HR for the patients in our breast cancer cohort in conjunction with all other known variables, including lymph node metastasis, TNM stage, and ER, PR, and ErbB2 positivity. To do so, we analyzed these variables using multivariate Cox Regression analysis. Patients with positive expression of miR-4728-3p had a decreased HR (Supplementary Table 1), however, the result was not statistically significant. To determine whether the expression levels of miR-4728-3p were correlated with the major molecular subtypes of breast cancer, including ErbB2-positive, ER-positive, PR-positive, and triple-negative cancers, we performed Pearson's chi-square analysis and found that miR-4728-3p expression was not significantly correlated with the major breast cancer molecular subtypes in our patient cohort (Supplementary Table 2). The lack of correlations in the multivariate Cox Regression analysis may due to the relatively small size of our cancer patient cohort, which may not be sufficient to achieve statistical significance.

To determine whether MST4 is overexpressed in human breast cancer, as may be expected if miR-4728-3p expression is lost, we examined the expression of MST4 in cancer *versus* normal adjacent breast tissue. We found that positive expression of MST4 was significantly higher (89.7% *n*=96/107) in cancer cases compared with normal tissues (33.3% *n*=6/18) (*P*<0.001). The positive rate of MST4 expression was also significantly higher among patients experiencing lymph node metastasis (96.2% *n*=63/65) compared with patients negative for lymph node metastasis (81.6% *n*=31/38) (*P*=0.012), indicating that MST4 may play an important role in breast cancer metastasis. Together with our *in vitro* results, these data indicate that MST4 may have potential as a novel therapeutic target in breast cancer. Overall, these patient sample study results strongly support the results from our *in vitro* and animal studies, demonstrating that mir-4728 is a tumor-suppressive miRNA that negatively regulates MAPK signaling through targeting MST4. In addition, these data indicate that miR-4728-3p may have value as a clinical prognostic biomarker for breast cancer patients.

## Discussion

Introns, although originally thought to be merely nonsense spacing elements in gene structure, have received extensive attention in recent years owing to the discovery of important functions for these sequences. Although some intronic miRNAs have functions unrelated to that of their host, others act in synergy with or antagonistically against the functions of their host genes.^20, 21^ Here, we discovered that mir-4728 functions in a manner indirectly antagonistic to the functions of its host gene, *ErbB2*. This complex mechanism of action represents a poorly studied mode of genetic regulation. Although mir-4728 is encoded within an intron of *ErbB2*, it does not directly target ErbB2 expression. Instead, it inhibits the ErbB2-downstream MAPK signaling pathway, which may partially explain how this miRNA suppresses the malignant behaviors of cancer cells. Therefore, this study represents an intriguing advance in our knowledge of gene regulation by miRNAs through providing strong evidence that an intronic miRNA can indirectly antagonize the canonical signaling of its host gene.

Extraordinarily precise control of genes is essential for cellular homeostasis and maintenance of a disease-free state. In the case of mir-4728, the miRNA may serve as an intrinsic protective mechanism by which cells may be able to delay transformation and lessen the metastatic potential of cells in which ErbB2 is activated. As loss of negative feedback control is a well-established mechanism by which malignancies develop, loss of tumor-suppressive miRNAs such as mir-4728 could cause oncogenic function in an otherwise still normally functioning cell, as the results of our human breast TMAs suggest.

As mir-4728 decreases the activity of ERK, a key downstream effector of ErbB2 signaling, mir-4728 may have potential in the treatment of ErbB2-overexpressing cancers, when delivery of miRNAs becomes a viable therapeutic option. In addition, we have demonstrated in this study that mir-4728 significantly inhibits the growth of ErbB2-negative MDA-MB-231 cells, as well as inhibiting the growth of ErbB2-moderate expressing MDA-MB-453 cells. This indicates that mir-4728's growth-inhibitory effects are not specifically dependent on the expression of ErbB2. Therefore, mir-4728 may also have potential in the treatment of cancers with ERK activation despite being ErbB2-negative. Given the important role of the MAPK pathway in tumor formation and development, the tumor-suppressive effects of mir-4728 are expected to have a broad spectrum of impact in multiple types of cancer. Further investigation of the role of this miRNA in other types of cancer is warranted.

There are likely other yet undiscovered targets of mir-4728 that need to be studied to fully explain the tumor-suppressive effects we observed. However, here, we have shown that the direct target MST4 plays a definitive role in cancer cell growth and motility. Although it has already been shown that MST4 plays a role in prostate cancer progression,^22^ to our knowledge, this is the first report that describes an important role for MST4 in breast cancer and shows that MST4 is overexpressed in breast cancer compared with normal breast tissues. Loss of miR-4728-3p expression during cancer development or progression may partially explain the increased expression of MST4 in cancer. Given the growth-promoting and migration-promoting effects of MST4 that we elucidated, this study may position MST4 as a new therapeutic target in breast cancer and generate interest in the role of MST4 in other human cancer types as well. Our findings have important implications for future experimental design when intronic miRNA-hosting genes are involved. For example, studies characterizing ErbB2 function that utilize an approach of overexpression or knockdown of ErbB2 by inserting or deleting only the gene-coding sequence are inherently flawed, given what we now know about mir-4728. The effects of the host gene and its intronic miRNAs must be evaluated together because the intronic miRNAs originating from those missing introns may have critical roles in positive or negative feedback with respect to the expression or function of the host genes or their downstream signaling pathways.

In summary, we have shown that mir-4728 negatively affects cell proliferation, migration, and invasion, while inducing apoptosis in breast cancer cells. Moreover, mir-4728 inhibits MAPK signaling through directly targeting the ERK upstream protein kinase MST4. In xenograft mouse models, exogenous expression of mir-4728 significantly decreases tumor formation and growth rate, whereas knockdown of endogenous miR-4728-3p increases the tumor growth rate. Importantly, the expression of miR-4728-3p is often lost in human breast cancer, which correlates with a worse overall patient survival, thereby demonstrating the potential prognostic value of this novel tumor-suppressive miRNA. Overall, these studies for the first time demonstrate a critical role for mir-4728 in the regulation of tumor growth and invasion/metastasis, and reveal a novel mechanism in which MAPK signaling is regulated in human breast cancer. This knowledge may significantly impact the diagnosis, monitoring, or treatment of breast cancer for patients in the future.

## Materials and Methods

### Cell lines and transfection

Human breast cancer cell lines MDA-MB-453, MDA-MB-231, SKBR3, and BT474, human cervical cancer cell line HeLa, and human ovarian cancer cell line SKOV3 were purchased from American Type Culture Collection (ATCC, Manassas, VA, USA). Cells were cultured in DMEM/F-12 (Mediatech Inc., Manassas, VA, USA) supplemented with 10% FBS and 1% Penicillin/Streptomycin. miRNA mimics and antisense miRNAs (anti-miRs) were purchased from Ambion (Grand Island, NY, USA). miR-mimic negative control #1 and anti-miR-negative control #1 (Ambion) were used as negative controls. Lipofectamine 2000 (Life Technologies, Grand Island, NY, USA) was used for transfection according to the manufacturer's instructions. Forty-eight hours after transfection, cells were harvested and prepared for the appropriate downstream assay.

### Phospho-kinase array

Human Phospho-MAPK Array kit was purchased from R&D Systems (Minneapolis, MN, USA) and was used per manufacturer's instructions with total protein lysate from MDA-MB-453 cells engineered to stably express either control vector or mir-4728 expression vector.

### Flow cytometry analysis

Cells of the indicated type were labelled using the Annexin V: FITC Apoptosis Detection Kit (BD Pharmigen, Franklin Lakes, NJ, USA) and analyzed using a FACSCanto Flow Cytometer (BD Biosciences, San Jose, CA, USA).

### Vector construction and establishing stable cell lines

Full-length human precursor mir-4728 together with 200 bp of flanking sequence was amplified from human genomic DNA and cloned into pMSCVpuro expression vector (Clontech, Mountain View, CA, USA). Primers used for the genomic amplification of mir-4728 were: 5′- TGACTCGAGCAGGAAGATCACTTGAGCCTAG and 5′- CGAGTTAACCCTCATTCTGTGGAGGAAGGAG with *Xho*I and *Hpa*I. Cells expressing control vector or mir-4728 overexpression construct were selected using puromycin. All cloned fragments were verified by sequencing. Infectious and replication-incompetent retroviral particles were produced according to manual protocol. MDA-MB-231 and MDA-MB-453 cell lines were infected with retroviral particles and 4 ug/ml polybrene to express mir-4728 or the puromycin resistance gene only (vector control). After retroviral infection and primary puromycin selection at 3 *μ*g/ml for MDA-MB-231 and 10 *μ*g/ml for MDA-MB-453, cell pools of MDA-MB-231/mir-4728, MDA-MB-231/Vector, MDA-MB-453/mir-4728, and MDA-MB-453/Vector were maintained for a maximum of 10 culture passages under lower puromycin concentration (1 *μ*g/ml). pLV-hsa-miR-4728-3p locker plasmid and corresponding control plasmid were purchased from Biosettia (San Diego, CA, USA). BT474-M1 cells were transfected with the pLV-hsa-miR-4728-3p locker plasmid or control plasmid expressing a scrambled sequence (vector control) using Lipofectamine 2000 (Life Technologies) according to the manufacturer's protocol. A GFP tag in the pLV-locker plasmid and control plasmid was utilized and BT474-M1/miR-4728-3p KD (knockdown) and BT474-M1/Vector cells were sorted on the basis of positive GFP expression using a BD FACS Aria II Cell Sorter. MST4 expression plasmid pCMV-Entry/MST4 and control vector pCMV-Entry were purchased from Origene (Rockville, MD, USA).

### Biological phenotype assays

Proliferation was assayed using direct cell counting every 24 h. Cell motility was assayed by standard scratch assay performed using an 18 h final time point. Invasion assay was performed using 24-well format and Boyden chambers coated with Matrigel matrix (BD Pharmigen). Briefly, 40 000 MDA-MB-231/mir-4728 or MDA-MB-231/Vector cells were seeded in the upper well in serum-free media. Cells that invaded through the Matrigel matrix and 8*-μ*m pore membrane over the course of 22 h were fixed with 4% paraformaldehyde, stained with 0.05% crystal violet, and counted under a light microscope.

### 3-D growth assay

Seventy microliters of Cultrex 3-D Culture Matrix reduced growth factor basement membrane extract (R&D Systems) were added to triplicate wells of eight Chamber Polystyrene Vessels (BD Falcon). The vessels were placed in a 37° 5% CO_2_ incubator for 30 min to allow the matrix to solidify. MDA-MB-231 cells (4000 per well) stably expressing vector control plasmid or mir-4728 overexpression plasmid were then seeded and allowed to grow for 8 days. The media was replaced with fresh growth media after 4 days.

### Caspase 3/7 assay

Caspase 3/7 activity was determined using 10 000 cells per well in a 96-well white-wall plate with the Caspase-Glo 3/7 kit from Promega (Madison, WI, USA) according to manufacturer's instructions.

### Quantitative real-time PCR (qPCR)

Total RNA was isolated from cultured cells using TRIzol reagent (Life Technologies). For miRNA expression analysis, qPCR was carried out using the qPCR miRNA Detection Kit and mirVana qPCR Primer Sets (Applied Biosystems, Grand Island, NY, USA) according to the manufacturer's protocols. Human U6 served as the internal control. Primers for miR-4728-3p: RT primer: 5′-GTCGTATCCAGTGCAGGGTCCGAGGTATTCGCACTGGATACGACCTGGGG; qPCR F 5′ -TTCCTCATGCTGACCTCCCTCCTG ; R 5′ – ATCCTGCCTCTCCTTCCTCCAC. All reactions were performed in triplicate. The relative amounts of miRNA were calculated by using the comparative CT method and presented as fold-change.

### siRNA experiments

siRNA oligonucleotide against MST4 was purchased from Sigma, with a scrambled siRNA (Sigma, St Louis, MO, USA) serving as a control. Transfection was performed using Lipofectamine 2000 Transfection Reagent according to the manufacturer's protocol. Forty-eight hours after transfection, the cells were prepared for the appropriate downstream assay.

### Luciferase reporter assay

The pMirTarget vector containing the MST4 3′ UTR clone or control non-targeting sequence were purchased from Origene. Deletion of the miR-4728-3p-binding site from the 3′ UTR of MST4, which is found at position 887–894 of the 3′ UTR, was accomplished *via* the QuikChange II kit (Agilent Technologies, Santa Clara, CA, USA) per manufacturer's instructions. Primers used to delete the miR-4728-3p-binding site were F: 5′-AGTACTAGTCCATGTGCCTTTTGGATCTT-3′ and R: 5′- AGTGTTTAAACATCATTGAGCTGCCTGGAAT-3′. MDA-MB-231, MDA-MB-453, and HeLa cells were co-transfected with pMirTarget-MST4, miR-4728-3p mimic, mimic negative control, and/or pMirTarget-Vector control as appropriate using Lipofectamine 2000. Twenty-four hours later, cells were harvested and lysed with passive lysis buffer (Promega, Madison, WI, USA). Luciferase activity was measured by using a dual luciferase reporter assay (Promega). The pRL-SV40 vector (Promega) was used as an internal control. The results are expressed as relative luciferase activity (Firefly Luc/Renilla Luc).

### Western blotting

Cells were harvested and lysed in NETN (20 mM Tris-HCl, pH 8.0, 100 mM NaCl, 1 mM EDTA, 0.5% NP40) for 20 min on ice. Lysates were cleared by centrifugation at 13 200 r.p.m. at 4 °C for 5 min. Supernatants were collected and protein concentrations were determined by the Bradford assay (Bio-Rad, Hercules, CA, USA). The proteins were then separated with a SDS-polyacrylamide gel and transferred to a nitrocellulose membrane (Bio-Rad). After blocking in TBS with 5% nonfat milk for 1 h, the membranes were incubated overnight at 4 °C with the primary antibodies in TBST with 1% BSA. The following antibodies were purchased from Cell Signaling (Beverly, MA, USA): p-ERK1/2, t-ERK, p-ErbB2, and tubulin. Antibodies were purchased from other companies as follows: *β*-actin antibody (Sigma), MST4 (Abcam, Cambridge, MA, USA), t-ErbB2 (Calbiochem, Billerica, MA, USA). Membranes were extensively washed with TBST and incubated with horseradish peroxidase conjugated secondary anti-mouse antibody or anti-rabbit antibody (dilution 1 : 2000, Bio-Rad). After additional washes with TBST, antigen–antibody complexes were visualized with the enhanced chemiluminescence kit (Pierce, Rockford, IL, USA).

### Animal study

All animals were maintained and all procedures performed according to a protocol approved by the Institutional Animal Care and Use Committee at the University of South Alabama. Female athymic nude mice (Harlan Sprague Dawley, Madison, WI, USA) were used. MDA-MB-231/Vector or MDA-MB-231/mir-4728 cells (2 × 10^6^) in 200 *μ*l of PBS and Matrigel (BD Biosciences) mixture (1 : 1) were injected subcutaneously into a mouse mammary fat pad. For BT-474M1/Vector or BT-474M1/miR-4728-3p KD, 0.72 mg 60-day release 17*β*-estradiol pellets (Innovative Research of America, Sarasota, FL, USA) were implanted subcutaneously. Two days later, 2 × 10^6^ cells were injected into the mammary fat pad. All tumor diameters were measured with digital micrometer calipers twice per week. The tumor volumes were calculated with the following formula: Volume (mm^3^) = *W*^*2*^ × *L*/2, where *W* and *L* are the minor and major diameters (in millimeters), respectively. At termination, the primary tumors were removed. Tumors were processed for subsequent protein and RNA analyses.

### TMAs and *in situ* hybridization

Human breast cancer TMAs were purchased from US Biomax (Rockville, MD, USA) and Novus Biologicals (Littleton, CO, USA). miRCURY LNA microRNA ISH Optimization Kit and miR-4728-3p Double-DIG labelled probe were purchased from Exiqon (Woburn, MA, USA), and *in situ* hybridization was performed according to the manufacturer's instructions. A semi-quantitative scoring criterion was used in which both the staining intensity and number of positive areas were recorded. A final staining index (scores ranging from 0 to 9) was used to quantify the total positive staining. To achieve the final staining index, the intensity of the positive staining (scores: negative=0, weak=1, moderate=2, strong=3) was scored. Then, the number of positive-stained cells (scores: <10%=1, 10–50%=2, >50%=3) was calculated. Finally, the intensity of staining (1–3) was multiplied by the score for the number of positively stained cells (1–3) to achieve the final staining score (1–9). The final scores were regarded as follows: 0=negative, 1=weak positive (score of 1, 2), 2=moderate positive (score of 3, 4), and 3=strong positive (score of 6–9). Overall survival was defined as the time of diagnosis to the date of death. The overall survival estimate over time was calculated using the Kaplan–Meier method and differences compared using the log-rank test. Results of analysis were considered significant in a log-rank test if *P*<0.05.

### Immunohistochemistry

Breast TMAs were purchased from US Biomax and Novus Biologicals and were immunohistochemically stained for p-ERK 1/2 (Cell Signaling) and MST4 (Abcam) using the streptavidin–biotin complex method from an anti-rabbit HRP-DAB Cell and Tissue Culture Staining Kit (R&D Systems). A semi-quantitative scoring criterion was used in which both the staining intensity and number of positive areas were recorded. A final staining index (scores ranging from 0 to 9) was used to quantify the total positive staining. To achieve the final staining index, the intensity of the positive staining (scores: negative=0, weak=1, moderate=2, strong=3) was scored. Then, the number of positive-stained cells (scores: <10%=1, 10–50%=2, >50%=3) was calculated. Finally, the intensity of staining (1–3) was multiplied by the score for the number of positively stained cells (1–3) to achieve the final staining score (1–9). The final scores were regarded as follows: 0=negative, 1=weak positive (score of 1–2), 2=moderate positive (score of 3–5), and 3=strong positive (score of 6–9).

### Statistical analyses

Statistical evaluation for data analysis was determined by Unpaired Student's *t*-test, unless otherwise indicated. All data are shown as the mean±standard error (S.E.M.). All experiments were performed with at least three independent replicates, and the data shown represent all available data. A *P*-value<0.05 is indicated by *P<0.01 by **P<0.001 by ***and *P*<0.0001 by ****.

## Figures and Tables

**Table 1:** Significant breast cancer prognostic factors determined by univariate analysis (Cox regression)

**Variables**	**Survival hazard ratio (95% CI)**	***P***
Lymph node metastasis (No/Yes)	2.698 (1.102–6.609)	0.030
TNM stage (II/III+IV)	4.934 (2.111–11.536)	<0.001
miR-4728-3p (+/−)	0.395 (0.161–0.967)	0.042

## Abbreviations

DAVID: Database for annotation, visualization and integrated discovery
DMEM: Dulbecco's modified eagle medium
DPBS: Dulbecco's phosphate buffered saline
ECAR: Extracellular acidification rate
ErbB: Erythroblastic leukemia viral oncogene
ER: Estrogen receptor
ERK: Extracellular-signal related kinase
FBS: Fetal bovine serum
HER2: Human epidermal growth factor receptor 2
HR: Hazard ratio
JNK: Jun N-terminal kinase
KD: Knockdown
KEGG: Kyoto Encyclopedia of Genes and Genomes
MAPK: Mitogen-activated protein kinase
miRNA: MicroRNA
MST4: Mammalian STE20-like protein kinase 4
OCR: Oxygen consumption rate
PBS: Phosphate buffered saline
PR: Progesterone receptor
qPCR: Real-time quantitative PCR
S.E.M.: Standard error of the mean
siRNA: Small interfering RNA
STE20: Sterile 20
UTR: Untranslated region
