# Supplementary figures and images for: ErbB2-intronic MicroRNA-4728: a novel tumor suppressor and antagonist of oncogenic MAPK signaling
Source: Cell Death Dis. 2015 May 7;6(5):e1742–. doi: 10.1038/cddis.2015.116 (PMC4669696; doi:10.1038/cddis.2015.116)

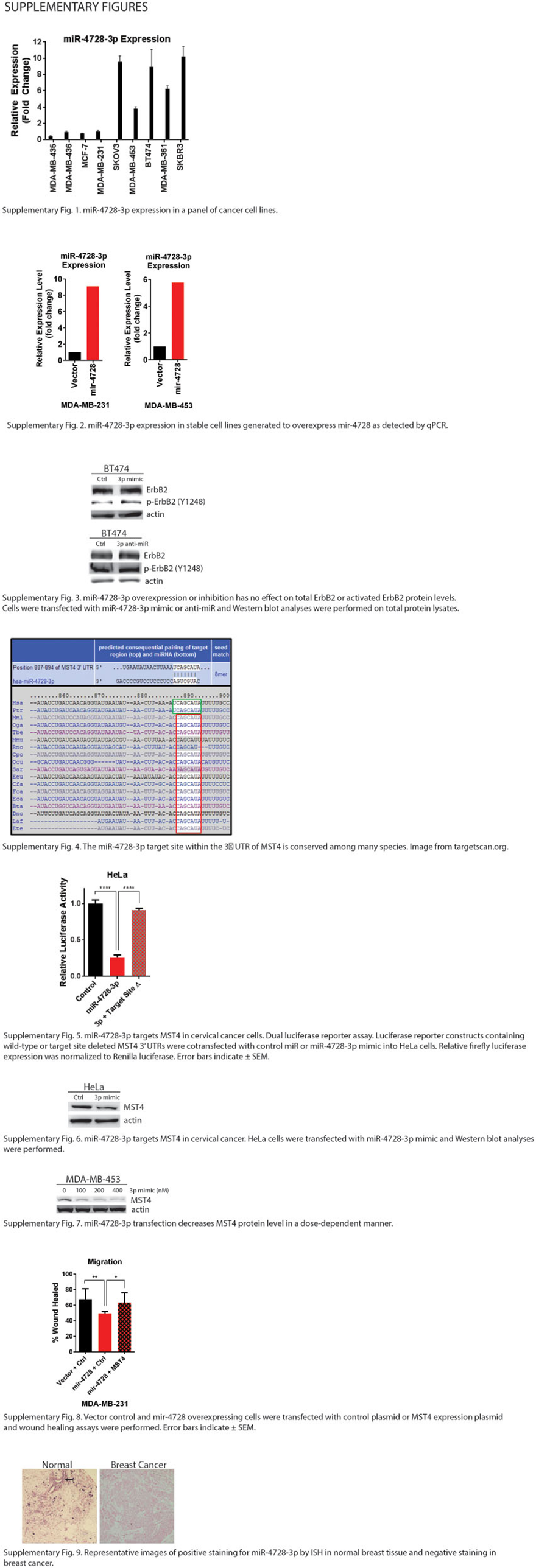

Supplement: Supplementary Information [file cddis2015116x1.tif]
