# Supplementary material for: ErbB2-intronic MicroRNA-4728: a novel tumor suppressor and antagonist of oncogenic MAPK signaling
Source: Cell Death Dis. 2015 May 7;6(5):e1742–. doi: 10.1038/cddis.2015.116 (PMC4669696; doi:10.1038/cddis.2015.116)
Supplement: Supplementary Tables [file cddis2015116x2.doc]

**Supplementary Table 1** **Prognostic factors determined by multivariate Cox Regression analysis.**

| **Variable** | **Survival**  **HR (95% CI) *P*** |
| --- | --- |
| Lymph node metastasis (No/Yes) | 0.337 (0.041-2.805) 0.871 |
| TNM stage (II/III+IV) | 11.040 (1.482-82.231) 0.062 |
| miR-4728-3p (+/-) | 0.530 (0.215-1.305) 0.535 |
| ER (+/-) | 0.555 (0.161-1.910) 0.350 |
| PR (+/-) | 0.518 (0.136-1.970) 0.335 |
| ErbB2 (+/-) | 0.453 (0.116-1.765) 0.254 |

**Supplementary Table 2 The relationship between miR-4728-3p and major breast cancer molecular subtypes.**

| **Characteristic** | **miR-4728-3p**  **+/-** | **miR-4728-3p**  **Positive Rate** | ***P*** |
| --- | --- | --- | --- |
| **ER**  **-**  **+** | 18/33  15/27 | 35.3%  35.7% | 0.966 |
| **PR**  **-**  **+** | 19/37  14/23 | 36.5%  37.8% | 0.700 |
| **ErbB2**  **-**  **+** | 20/24  13/36 | 45.5%  26.5% | 0.057 |
| **Triple Negative** |  |  |  |
| **Yes**  **No** | 14/20  23/67 | 41.2%  25.6% | 0.090 |
